# Lung Transplant Go (LTGO): A Randomized Controlled Trial to Evaluate the Efficacy of a Telerehabilitation Behavioral Exercise Intervention after Lung Transplantation

**DOI:** 10.63144/ijt.2026.6734

**Published:** 2026-06-01

**Authors:** Christopher C. Imes, Frank C. Sciurba, Andrea L. Hergenroeder, Dianxu Ren, Elizabeth A. Lendermon, Bryan Willey, Alice Curtis Cline, Seol Ju E. Moon, Melissa Vendetti, Kristen Jones, Haydar Al Ebousi, Annette Devito Dabbs

**Affiliations:** 1 University of Pittsburgh, School of Nursing, Pittsburgh, Pennsylvania, USA; 2 University of Pittsburgh, Department of Medicine, Pittsburgh, Pennsylvania, USA; 3 University of Pittsburgh, School of Health and Rehabilitation Sciences, Pittsburgh, Pennsylvania, USA; 4 VA Pittsburgh Healthcare System, Pittsburgh, Pennsylvania, USA; 5 University of Minnesota, School of Nursing, Minneapolis, Minnesota, USA

**Keywords:** Exercise interventions, Lung transplantation, Randomized controlled trial, Telerehabilitation

## Abstract

The Lung Transplant GO (LTGO) randomized controlled trial evaluated the efficacy of a telerehabilitation behavioral exercise program after lung transplantation compared to enhanced usual care (EUC). We hypothesized that the LTGO treatment condition would show significantly greater improvements in change scores from baseline to 3 and 6 months for outcomes of physical function, physical activity, and blood pressure control compared to the EUC condition. The sample included 88 lung transplant recipients (LTRs) randomized 1:1 to either LTGO or EUC. No statistically significant differences in change scores for physical function, physical activity, and blood pressure control were found between treatment groups at 3 and 6 months. While no statistically significant difference in outcomes were found between groups, null findings are not uncommon. These findings and the discussion of post-trial considerations are important for the design of future trials to inform the field of telerehabilitation.

Lung transplantation is an established treatment option for individuals with advanced lung disease to improve survival and quality of life (Valapour et al., 2025). Lung transplant recipients (LTRs) report improvements in health compared with pretransplant levels (Kolaitis & Singer, 2018). However, limited exercise capacity often persists after transplant (Bartels et al., 2011; Rozenberg et al., 2014; Walsh et al., 2013), which undermines the intended benefits of the highly selective, resource-intensive transplant procedure (Kobashigawa et al., 2019). Exercise is also known to aid in the control of hypertension, a common side-effect of immunosuppressants (DeVito Dabbs & Song, 2008). Based on evidence for the benefits of pulmonary rehabilitation (PR) to improve functional exercise capacity in persons with end-stage lung disease, including LTRs (Rochester et al., 2015; Wickerson et al., 2015), it is prescribed after lung transplantation to optimize functional status (Abidi et al., 2023; Gutierrez-Arias et al., 2021). Yet, despite its potential benefits, due to multiple barriers, such as lack of access to local PR programs, low start and completion rates (especially for people living in rural areas with limited incomes who lack insurance coverage for PR), lack of transportation, scheduling issues, or disruption of usual routines (Fischer et al., 2009; Jones et al., 2017; Spruit et al., 2014), uptake and completion rates of in-person PR are alarmingly low (Lahham & Holland, 2021).

Telerehabilitation (TR) is defined as the “application of information and communications technology to deliver rehabilitation services over a distance by linking a healthcare provider to a beneficiary, caregiver, or any person(s) responsible for delivering care to the beneficiary, for the purposes of screening, assessment, intervention, consultation, coaching, or supervision and monitoring” (Hill et al., 2025, p. 3). Many studies suggest that TR is cost-effective and associated with participant satisfaction (Dennett et al., 2021; Hansen et al., 2020; Subedi et al., 2020), presumably because it reduces many of the barriers to in-person PR. Studies of remote options for delivering PR have shown promise (Chan et al., 2016; Holland et al., 2021; Singer et al., 2018), but the trials to date have been small, retrospective, or lacked comparison groups (Bonnevie et al., 2021; Myers et al., 2021). Furthermore, the active intervention periods were brief (less than 3 months) and did not include a maintenance phase or the behavioral change strategies that are known to help sustain the benefits of a formal exercise training program (Michie et al., 2009).

We posited that an alternative delivery model for PR was needed to overcome challenges of in-person programs that limit the initiation and adherence of LTRs to exercise. We conducted a randomized controlled trial (RCT) to evaluate the efficacy of Lung Transplant Go (LTGO), a behavioral telerehabilitation exercise intervention to improve physical function, physical activity, and blood pressure control after lung transplantation. The study was approved by the Institutional Review Board (19020357) and registered in ClinicalTrials.gov (NCT03728257).

## Methodology

### Research Design

The study was a single-site, 2-group RCT with LTRs randomized 1:1 to either the LTGO intervention group or to the enhanced usual care (EUC) group. We used the intent-to-treat (ITT) approach for the analyses wherein all LTRs were analyzed in the groups to which they were randomly assigned, regardless of treatment received or protocol deviations (McCoy, 2017). We hypothesized that LTRs allocated to LTGO treatment would show significantly greater improvements in physical function, physical activity, and blood pressure control from baseline to 3 months and baseline to 6 months compared to LTRs allocated to the EUC treatment. Exercise self-efficacy and adherence to self-monitoring would mediate the intervention effects. A variety of sociodemographic and clinical factors would moderate the intervention effects.

### Participants

LTRs were recruited from a large lung transplant program affiliated with an academic tertiary medical center in the Mid-Atlantic region of the U.S. Consecutive sampling was employed to prescreen the electronic health records of all LTRs transplanted between April 2019 and March 2023 to determine inclusion and exclusion criteria with the transplant physician (Table 1). Informed consent conversations occurred in person or via Zoom (San Jose, CA), a Health Insurance Portability and Accountability Act (HIPAA)-compliant 2-way video-conference platform (Centers for Disease Control & Prevention, 2024). Zoom protects and encrypts all audio, video, and screen sharing data, without access to identifiable personal health information.

### LTGO Treatment

A structured exercise progression protocol developed for LTGO (Hergenroeder et al., 2023) was based on the American Association of Cardiovascular and Pulmonary Rehabilitation Guidelines (Collins et al., 2014). The interventionist followed a replicable approach to prescribe and advance the exercise prescription according to each LTR’s baseline fitness level and progress. Walking was the primary physical activity prescribed; however, other forms of physical activity were considered acceptable alternatives. Structured exercises included strength, balance, and flexibility training, and gradually increased targets for steps per day. Phase 1 included weekly sessions with the interventionist that included individualized, supervised exercise training for 12 weeks plus eight behavioral coaching sessions using the HIPAA-compliant Zoom platform. Additionally, participants were advised to follow their exercise prescription at least 1 time per week on their own. Phase 2 (maintenance) included monthly telephone calls with behavioral coaching for 12 weeks to promote the transition to successful self-management. The addition of the maintenance phase, guided by an individualized behavioral contract, was designed to enhance LTRs’ ability to sustain self-management of exercise with waning support of an interventionist overtime.

### Enhanced Usual Care Treatment

EUC for LTR included encouragement by the transplant team to participate in structured exercise activities (U.S. Department of Health and Human Services, 2018). The EUC group was also provided with a Fitbit to monitor steps per day and given a printed instruction manual developed for the LTGO study which explained how to install and use the activity tracker. LTRs in the EUC group also received six monthly newsletters on health topics related to lung transplant (e.g., food safety, environmental health, flu, mental health, skin care and oral health).

### Study Procedures

Early in the LTGO trial, to comply with the infection mitigation strategies for COVID-19, all study activities were adapted to be performed remotely, via HIPAA-compliant Zoom, for both groups, including recruitment, consenting, intervention delivery, and outcome assessments according to previously reported protocol modifications (Moon et al., 2021). LTRs underwent a baseline assessment to measure sociodemographic and clinical characteristics to establish pre-intervention levels for all outcomes. The statistician randomized LTRs 1:1 to either the LTGO or EUC group, using a blocked randomization scheme stratified by sex, and length of transplant-hospital stay (≤2 weeks or >2 weeks), to achieve balance between groups for known differences in exercise capacity and hospital-associated deconditioning. We projected enrolling 112 subjects a priori to reach a final sample of 80 randomized subjects (40 per group) to achieve 80% power to detect an effect size as small as 0.64, α=0.05, two-tailed, for measures of physical function (physical well-being, balance, lower body strength), physical activity (steps per day and time spent in moderate and vigorous activity), and blood pressure control measured at baseline, 3 and 6 months following the intervention.

Outcome assessors were blinded to group assignments. Data collectors were trained to perform study assessments until they demonstrated competency. Quality control checks of inter-rater reliability between assessors were repeated every quarter and remediation was instituted to remedy any drift from the assessment protocol less than (r = .97). Assessors were trained to abstract data from the electronic health record (EHR) using established code books. Inter-rater reliability was determined between two raters for every 10 records. Performance of all assessments and delivery of intervention sessions and phone calls were audio or video recorded. Intervention fidelity was monitored for 10% of phase 1 exercise sessions per LTR and all phase 2 phone calls. Intervention fidelity was 97.2 % for phase 1 and 97.6% for phase 2 sessions. Data safety monitoring meetings were held monthly with the principal investigator (PI) and project director (PD), and annually with PI, PD, and the medical monitor.

The interventionist maintained an attendance record for each of the 12-weekly supervised LTGO exercise and eight behavior coaching sessions per LTR during phase 1, and attendance for the 3-monthly intervention phone calls assessed at the end of phase 2. The degree of adherence was determined by summing the number of weekly supervised exercise sessions and behavior control topics attended during phase 1, and the number of monthly phone calls completed during phase 2. The higher the sum, the greater the participation in the program.

A full description of the RCT (Vendetti et al., 2023), exercise protocol (Hergenroeder et al., 2023), and remote assessment protocols (Moon et al., 2021) were published previously. The outcome measures are summarized in Table 2 with details described below.

### Primary Outcome Measures: Physical Function

#### The St. George Respiratory Questionnaire (SGRQ; Jones et al., 1991; Jones, 2002; Jones et al., 2014)

The SGRQ, a 2-part, 50-item, self-report questionnaire, was administered at baseline, 3 and 6 months in person or by phone to assess overall respiratory health, daily life, and perceived well-being. Lower scores indicate better functioning. Empirical evidence suggests that a mean change in total score of > 4 units between baseline, 3, and 6 months is considered a minimum clinically important difference.

#### Berg Balance Scale (Berg, 1992; Berg et al., 1995; Blum & Korner-Bitensky, 2008)

The Berg Balance Scale is a reliable assessment tool to test an individual’s ability to perform tasks that require balance (e.g., sitting-to-standing, placing alternate foot on stool) at baseline, 3 and 6 months. To complete the Berg Balance scale, the LTR was instructed to perform 14 specific tasks related to dynamic and static balance performed while standing, sitting, or making simple movements while being observed and scored by a trained assessor. Higher scores indicate better functioning.

#### The 30-second Sit-to-Stand (30-s STS; Benton & Alexander, 2009; Jones et al., 1999)

The 30-s STS was used to measure lower body strength at baseline, 3, and 6 months. LTRs were instructed to sit straight up in an armless, 17-inch height chair with their arms crossed, feet flat on the ground, and rise to a full standing position and return to a sitting position as many times as they could in 30 seconds. The higher the number of sit-to-stand repetitions, the better the lower extremity strength and endurance (Zanini, et al., 2015). Multiple studies found that the 30-s STS was moderately correlated with the Six-Minute Walk Test, including correlations of r = 0.53 to 0.66 (p < .001) in healthy young adults (Gurses et al., 2018), adults with chronic obstructive pulmonary disease (Zhang et al., 2018), and pulmonary hypertension (Ozcan Kahraman et al., 2020).

### Primary Outcome Measures: Physical Activity

#### Walking

All LTRs were provided a Fitbit Charge 3 (Fitbit Inc., San Francisco, CA), a wearable sensor that recorded steps throughout the day. LTRs were instructed to wear the sensor on their nondominant wrist at least 8 hours per day. Walking was measured as the average steps per day for 14 days at baseline, 3, and 6 months (Noah et al., 2013; Strath et al., 2013).

#### Time Spent in Moderate or Vigorous Activities (Masse et al., 2005; Van Remoortel, 2012)

All LTRs were given an Actigraph GT3X (ActiGraph, LLC., Pensacola, FL) with an accelerometer to measure physical activity for a 7-day period at baseline, 3 and 6 months. LTRs were instructed to (1) wear the Actigraph on their waist during waking hours (except during imaging studies such as bronchoscopy, bathing or showering), (2) keep a paper activity diary, and (3) return the device and diary by mail in a pre-paid envelope. The Actigraph provided tri-axial vector data in activity units, metabolic equivalent tasks (METs), or kilocalories. Validity has been established in people with chronic lung disease. Physical activity was reported as average minutes spent per day in moderate/vigorous (≥3 METs) activity at each time point.

### Secondary Outcome Measure

#### Blood Pressure Control (Flack & Adekola, 2020)

Blood pressure control was measured as a change in stage of hypertension between baseline and 3 and 6 months. Systolic and diastolic blood pressures were abstracted from the EHR, then categorized from 1 (normal) to 4 (stage 3) according to the standard categories for hypertension. Blood pressure control was reported as the proportion of LTRs whose BP status improved, stayed the same, or worsened at each time point.

### Potential Moderators

#### Dyadic Adjustment Score (DAS; Spanier, 1976)

The DAS is a 15-item, Likert-scale, self-report questionnaire for LTRs to rate the quality of the relationship with the adult whom they identify as most involved in assisting with his/her daily care. Although spouses (generally wives) usually fulfill this caregiver role, the scale also has been found to be applicable for assessing the supportive nature of non-spouse types of LTR-caregiver dyads (DeVito Dabbs et al., 2013). Higher scores reflect higher relationship quality which could influence the target outcomes.

#### Charlson Comorbidity Index (CCI; Bernardini et al., 2004; Charlson et al., 1994)

The CCI was used to assess the presence of up to 19 comorbid conditions abstracted from the EHR. The CCI is scored by summing the assigned relative risk scores for each condition based on the evidence-based prediction of 1-year mortality. The higher the total score, the greater the number and severity of comorbidities which could influence the target outcomes.

#### Questionnaire for Lung Transplant Patients (QLTP; DeVito Dabbs et al., 2004)

The QLTP is a self-report symptom checklist comprised of 66 symptom items. A score of 1 is assigned for each symptom experienced in the past 2 weeks. The scores for each item are summed to compute the total score, ranging from 0 to 66. The higher the score, the greater the symptom burden which could influence the target outcomes.

#### Symptom Checklist 90-Revised (SCL-90-R; Derogatis, 1994)

Psychological distress was measured using the Anxiety and Depression Subscales of the SCL-90. These self-report subscales measure the severity of symptoms related to anxiety and depression during the prior two weeks. Items are rated on a five-point Likert-type scale (0 = not at all to 4 = extremely distressed). Subscale scores are computed by averaging items. For the current analyses, the sub-scale scores were used to identify the proportion of LTRs reporting any clinically significant symptoms of psychological distress, defined as any score exceeding one standard deviation above the gender-specific normative mean on either the anxiety or depression sub-scale (DeVito Dabbs et al., 2003). Higher scores reflect higher distress, which could influence the target outcomes.

#### Pittsburgh Sleep Quality Index (PSQI; Buysse et al., 1989)

Sleep quality was measured using the PSQI, a 19-item, self-report scale to assess sleep during the previous month. The PSQI measures seven components: sleep duration, sleep disturbances, sleep quality, daytime dysfunction due to sleepiness, sleep efficiency, sleep latency, and use of sleep medications. The range of possible total PSQI scores is 0 and 21. For the current analyses, total PSQI scores were used to identify which LTRs reported poor sleep quality using the established cut-off score >5. Higher scores reflect poor sleep quality, which could influence the target outcomes.

### Potential Mediators

#### Adherence to Self-Monitoring

The percentage of days that the LTR wore the Fitbit over a 14-day period was used to measure the adherence to self-monitoring of walking goals as a potential mediator based on evidence that performing self-monitoring is an effective behavior change technique for achieving goals (Michie et al., 2009). A minimum of at least 150 steps per day was required to count as a day used. Higher scores indicate higher levels of adherence (Strath et al., 2013).

#### Self-Efficacy to Regulate Exercise (SERE; Bandura, 2006)

The SERE, an 18-item, self-report scale, was used to quantify perceived capacity to perform the recommended exercise routine regularly (≥3 times/week) as a potential mediator. Each item of the SERE asked the LTRs to rate how certain they feel about performing the exercise routine in various situations. Strength of efficacy beliefs are rated on a 100-point scale (0 = cannot do to 100 = highly certain can do) and averaged to calculate a score (range: 0–100). Higher scores indicate higher levels of self-efficacy. Self-efficacy has been identified as an important determinant of future health behavior and health behavior change (Holloway & Watson, 2002). The SERE was deemed reliable and valid in measuring significant improvements in a wide range of regulatory behaviors and populations (Oaten & Cheng, 2006).

### Data Management and Analysis

Longitudinal data were collected and managed using Research Electronic Data Capture (REDCap) software (Harris et al., 2009) tools hosted at the University of Pittsburgh. All REDCap data were exported to SPSS (IBM v 27.0) and R (R Core Team v 4.4.3). All statistical analyses were conducted in SAS (SAS Institute Inc. v 9.4) and R (R Core Team v 4.4.3). Study 360 (Engberg et al., 2006) was used to track study due dates and communicate with LTRs. Assumptions were checked, including normality and equality of variances.

Initial analyses focused on characterizing the study sample and distribution of key measures using Wilcoxon rank sum tests for ordinal variables, Pearson’s chi-squared test for categorical variables, and Fisher’s exact test for categorical variables with small cell sizes. Descriptive statistics were conducted for sample demographics, and to observe the distribution of all primary and secondary outcome measures at each time point: baseline (0-months), 3-months, and 6-months in addition to changes in scores from 0–3 and 0–6 months. Bivariate analyses were conducted to explore associations between covariates (sociodemographic and clinical characteristics) and primary and secondary outcome measures. Analyses of the raw values included all participants with data for the given measure and timepoint. The analyses comparing change scores between 0–3 and 0–6 months included only participants with data at both time points.

The primary analysis to compare the effect of the LTGO intervention to EUC involved examining the association between treatment assignment and change scores for all primary and secondary outcome measures, from 0–3 months and 0–6 months. A repeated measures mixed-effects regression model was utilized for this analysis, with fixed effects for the predictor variables (time and treatment group assignment), and a random intercept for LTR as measure values within each participant are related to one another but differ between LTRs. The analyses, using a linear mixed-effects model for numeric variables, and a generalized linear mixed effects model for blood pressure (the only categorical outcome variable) were conducted using R package lme4 v 1.1–37 (Bates et al., 2015). These models are robust for handling longitudinal data with missing values and account for the correlated nature of repeated measurements within individuals and included all participants. For blood pressure, a categorical outcome variable, confidence intervals for the fixed effects were calculated using parametric bootstrapping with 1,000 samples. Missing values were handled in the model by maximum likelihood estimation, and no imputation was conducted.

The mixed effects model is expressed here:

Yij = β0+ β1 × groupj + β2 × timeij + β3 × (groupj × timeij) + uj + εijYij = *β*0+ *β*1 × groupj + *β*2 × timeij + *β*3 × groupj × timeij + uj + *ɛ*ij

Where:

Yij = the value of the dependent variable for participant j at time i
β0 = the intercept for Y when all other variables remain constant (fixed intercept)
β1 = the change in Y based on group assignment (fixed coefficient)
β2 = the change in Y based on time point (fixed coefficient)
β3 = the interaction between time and group assignment uj = the intercept for subject j (random intercept)
group = treatment group, EUC or LTGO (fixed predictor variable)
time = time point of measurement for the i-th measurement of participant j, baseline, 3-months, and 6-months (fixed predictor variable)
*ɛ*ij = residual error for the i-th observation of the j-th subject. This captures the unexplained variance in Y after accounting for the fixed and random effects

Further analyses were performed to examine whether any sociodemographic characteristics moderated the effect of the intervention on the outcomes and whether self-efficacy to regulate exercise and self-monitoring steps per day mediated the relationship between treatment assignment and outcomes.

## Results

### Sample Characteristics

**Figure 1:**
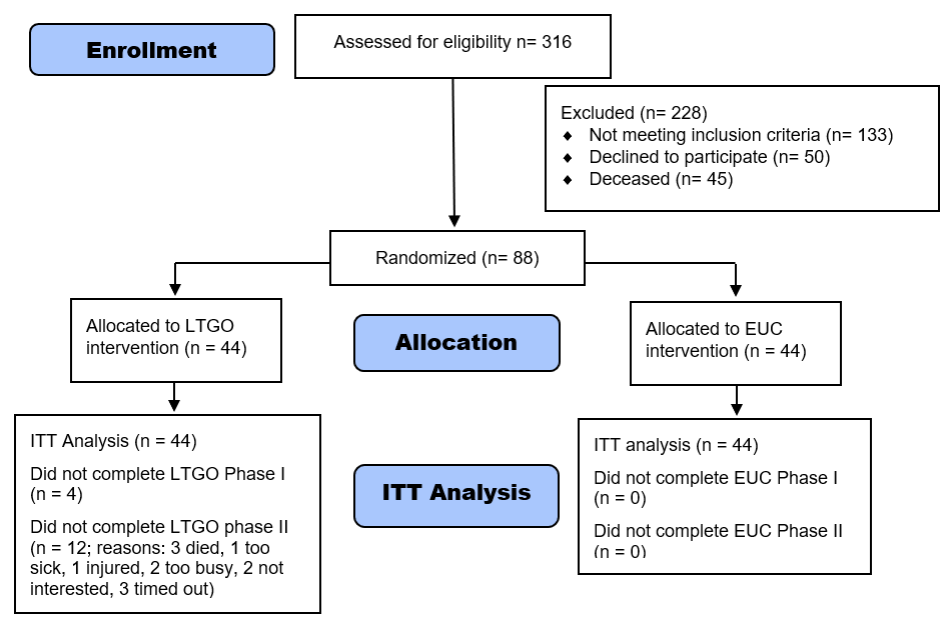
CONSORT Diagram

Eighty-eight LTRs were enrolled, 44 randomized to LTGO and 44 randomized to EUC. The Consolidated Standards of Reporting Trials (CONSORT) diagram for documenting the flow of participants through the LTGO trial is shown in Figure 1 (Hopewell et al., 2025). Characteristics of the sample are shown in Table 3. The mean age of the sample overall was 56 years, with a range of 21–73 years. Forty-eight LTRs were male and 40 were female. The majority of LTRs were partnered (72%, n = 63) and White (90%, n = 78). The majority completed some college education or more (56%, n = 49) and were not working (95%, n = 76). Income levels varied considerably, with 16% (n = 13) earning less than $20,000 annually, 20% (n = 16) earning more than $100,000, and the rest in between. There were no statistically significant between-group differences in baseline characteristics reflecting equivalency at baseline. There were no unanticipated adverse events.

### Outcome Measures

The raw values for primary and secondary outcomes per assessment time point show very little variation between treatment groups (Table 4.) Physical function measured by sit-to-stand scores remained stable between time points. Berg balance scores and SGRQ scores improved from baseline to 3- and 6-month assessment points for both groups (LTGO and EUC). In the interval from baseline to 3-months, the LTGO group reported a 6-point reduction in SGRQ scores while the EUC group reported a 5-point reduction, with a mean difference of 1.81 (p = 0.452, 95% CI −2.95, 6.57). In the longer treatment interval from baseline to 6-months, the LTGO group reported a 7-point reduction in SGRQ scores compared to 6-point reduction in the EUC group, with a mean difference of 0.5 (p = 0.826, 95% CI −3.99, 4.98).

Change scores from baseline to 3- and 6-month assessments for physical function, physical activity, and blood pressure control were not significantly different between treatment groups (Table 5). We examined the partial results of the linear mixed effects and generalized linear mixed effects models for the primary and secondary outcomes. For all variables, group assignment was found to be non-significant. Due to null findings for treatment effects, mediation analysis was not conducted.

Results of the unadjusted mixed-effects regression model for the effect of treatment group on outcome measures are shown in Table 6. None were statistically significant with p values between 0.06 to 0.93. Both treatment groups had statistically significant improvement in scores from 0–3 months and 0–6 months, but the interactions between group and interval were not significant.

### Subgroup Analysis

The raw values for potential moderators and mediators per assessment time point by treatment group are shown in Table 7. There were too few minorities to examine subgroup differences for race and ethnic subgroups. Moderation was not explored since the unadjusted model was not significant. Due to the non-significance of between-group differences in change outcomes the planned analysis of potential mediation was not performed.

However, exploratory post-hoc analyses were conducted to assess whether the intervention effect differed among LTRs with higher rates of adherence to self-monitoring activity and self-efficacy to regulate exercise. These were calculated by selecting those LTRs with the highest quartiles of adherence to self-monitoring using the mean percentage of daily Fitbit use at 3- and 6-month time points, and LTRs with the highest quartile of self-efficacy scores using the mean SERE scores at baseline, 3- and 6-month time points. There were nine LTRs in the highest quartile of mean adherence rates (only 36 LTRs had Fitbit adherence reported at any time point), and 23 LTRs in the highest quartile of SERE scores (all LTRs had a SERE score at baseline, and there was a tie at numbers 22 and 23, so both were included). Treatment effects among subgroups with scores in the highest quartile of adherence to self-monitoring and SERE using the same models as for the primary outcomes are shown in Table 8. Among LTRs with scores in the high-adherence to self-monitoring subgroups, the LTGO intervention was significantly associated with improvements in Berg Balance Scores, indicating improvement in balance compared to participants in the EUC group. Other outcomes were non-significant, with p values between 0.22–0.90. Given the small sample size, these results should be interpreted with caution.

### Completion and Acceptability of LTGO

In phase 1, LTRs participated in a mean of 9.5 out of 12 (79%) weekly exercise training sessions and 7.5 of the 8 (93%) behavioral coaching sessions. During phase 2, participants completed a mean of 2.2 of the 3 (74%) monthly phone calls.

Findings from post-intervention semi-structured interviews conducted with LTRs randomized to the LTGO group were generally positive with reported benefits including motivation, building strength, and becoming more physically active (Jena et al., 2025). Participants mentioned the program’s convenience, individualized supervision, and behavior change techniques as positive aspects of the intervention. Any reported frustrations with the intervention technology or delivery were few and resolved promptly.

## Discussion

For LTRs in both treatment groups, scores on most outcome measures at each time point were equivalent, but for some outcomes, the mean scores improved and exceeded the minimum clinically important difference. However, the hypotheses for this study were not supported based on the absence of any statistically significant between-group differences in change scores for any outcomes between baseline to 3 and 6 months. ‘Null’ findings are not only important to report, but the data collected from trials (even with ‘null” findings) offers the opportunity to gain a better understanding of the mechanisms that may explain the relationships between the interventions and outcomes.

We considered a myriad of limitations and design choices made in the course of developing and testing the LTGO intervention (Abadie, 2020), as possible explanations for the failure to detect statistically significant improvements in the primary and secondary outcomes between baseline and 3 and 6 months for the LTGO group compared to the EUC group.

First, there may have been a mismatch between the theory, intervention, and the problem. For instance, the LTGO telerehabilitation exercise intervention was designed to overcome some of the barriers to completion of in-person pulmonary rehabilitation programs, such as inconvenient scheduling, the need to travel from home, and lack of 1:1 customized exercise training sessions, but there may be other unknown barriers that were not addressed. The LTGO intervention also integrated evidence-based behavior change techniques known to promote attainment of exercise goals such as goal-setting and self-monitoring of behavior (Michie et al., 2015), but there may be other tacit factors impeding behavior change that were not directly addressed.

Second, the intensity (strength, frequency, or dosage) of the LTGO intervention may have been too low to achieve the intended outcomes of interest within the duration of the study period. While the LTGO exercise progression model was customized to the needs of each LTR, the incremental increases in intensity may have been too small or too infrequent to achieve demonstrable training effects. Furthermore, one supervised exercise session with the interventionist per week, plus one session per week on their own, may not have been frequent enough for the LTRs to achieve the recommended activity goals.

Third, the LTGO intervention may not reflect a large enough enhancement over EUC. However, taking the LTGO intervention at face value, LTGO offered more structure and supervised exercise and coaching sessions beyond the mere encouragement of the transplant team to participate in structured exercise activities, the Fitbit and newsletters that the EUC received. All participants entering the study may have been highly motivated to augment their activity level, and those assigned to the EUC group may have increased their activity even with our minimal interface and encouragement. The increase of several outcome metrics in both groups support this possibility. Further exploration is needed to determine assumptions regarding the relative value of the elements of the LTGO intervention compared to EUC.

Fourth, some outcome measures may not have been as sensitive to change as others. For example, early in the trial period we were forced to abandon two in-person, objective outcome measures, namely the Cardio-Pulmonary Exercise (Porszasz et al., 2003), a test of maximal exercise capacity performed in a laboratory setting, and the Six-Minute Walk Test (American Thoracic Society, 2002), a well-validated measure of functional capacity, to comply with restrictions during the COVID pandemic. The measures that were included may have been less precise measures of true change (Sprague, 1995).

Fifth, it is possible that the study procedures were not standardized, protocolized or implemented reliably with high enough fidelity. However, our quality control documentation provided evidence that we met the conventional metrics for reliability, intervention fidelity, and replicability of all study procedures, making this explanation unlikely.

Sixth, the COVID pandemic may have contributed to the results. Overall, the pandemic was associated with a decrease in physical activity and an increase in sedentary behaviors (Park et al., 2022). It is likely the pandemic’s effect on LTRs was similar. Given the excess hospitalizations and mortality of COVID-affected solid organ transplant recipients (Kolla et al., 2023; Yamanaga et al., 2024), LTRs’ fear of a COVID infection could have altered participant’s physical activity routines as well as access to exercises spaces. Among LTRs, steroids were associated with a high risk of COVID-related hospitalization (Kolla et al., 2023). Hospitalization, especially one for a respiratory infection, would have negatively impacted the exercise capacity gains achieved with PR. While these barriers would have affected individuals in the LTGO and EUC groups, it is unknown if the impact was equal between the group’s members.

Finally, the most likely explanation was limited statistical power to detect treatment effects. While we met our projected enrollment goals with a final sample of 88 randomized subjects based on the intent to treat approach, 44 LTRs were analyzed per group. However, due to missing values, the number of observations LTRs contributed for each outcome measure was less than projected, therefore the observed impact may not have been large enough. If the trial had a larger final sample or a more complete data set, the differential treatment effects may have been detected more reliably.

## Conclusions

The LTGO intervention was feasible, acceptable, safe and well-tolerated, but it did not demonstrate superiority over EUC on the prespecified outcomes in this sample. None of the possible explanations for the null findings were due to issues with telerehabilitation delivery platforms, therefore remote options for delivering pulmonary rehabilitation continue to hold promise. Because the actual mean daily steps per day and time spent in moderate or vigorous activity for both treatment groups were lower than current recommended levels for adults (Centers for Disease Control & Prevention, 2023; Paluch et al., 2022), there is still a need for interventions to promote better exercise behaviors for LTRs. The null findings and the post-trial considerations described here are important lessons for future researchers and clinicians involved in the design and evaluation of telerehabilitation interventions trials.

## Figures and Tables

**Table 1:** Study Eligibility Criteria

Inclusion Criteria	Exclusion Criteria
Underwent a lung transplant procedure (including re-transplant) ≥ 18 years of age Able to read and write English >4 weeks post lung transplantation surgery Discharged home after lung transplant surgery MD report of difficulty walking .25 miles or climbing 10 steps without resting Transplant physician confirms patient eligibility	Underwent a multi-organ transplant Declined to be asked screening questions, or declined an introduction to the research team to hear about research Medical issue precluding participation Other chronic conditions that may severely limit participation in exercise training, e.g., cardiac, musculoskeletal, or cognitive impairments No home internet or smart device with Bluetooth capabilities Concurrent participation in a formal exercise program, e.g., pulmonary rehabilitation, during the active eligible study period, with no plans to stop formal exercise >18 months post-transplant hospital discharge (time/scheduling delays, transportation issues, etc.)

*Note*. Adapted from Vendetti et al., 2023. Re-use attribution: https://creativecommons.org/licenses/by-nc-nd/4.0/

**Table 2:** Summary of Measures

Variables	Variable description	Measures	Time of Measurement		
			Base	3M	6M
**Primary Outcomes**					

Physical function	Physical performance and wellbeing	St. George Respiratory Questionnaire (Jones et al., 1991; Jones, 2002)	X	X	X
	Balance	Berg Balance Scale (Berg, 1992; Berg et al., 1995; Blum & Korner-Bitensky, 2008)	X	X	X
	Lower body strength	30-Second Chair to Stand (Benton & Alexander, 2009; Jones et al., 1999)	X	X	X
Physical activity	Walking	Average Steps per day for 14 days (Noah et al., 2013; Strath et al., 2013)	X	X	X
	Minutes per day spent in moderate and/or vigorous activities	Actigraph GT3X for 7 Days (Masse et al.,	X	X	X
		2005; Van Remoortel et al., 2012)			

**Secondary Outcome**					

BP Control	BP Stage (improved, same or worsened)	ACC/AHA Blood Pressure Guidelines (Flack & Adekola, 2020)	X	X	X

**Potential Mediators**					

Self-monitoring	Adherence to self-monitoring steps per day	% of days with Fitbit ≥150 steps per day (Noah et al., 2013; Strath et al. 2013)	-	X	X
Self-efficacy	Exercise self-efficacy	Self-Efficacy to Regulate Exercise (Bandura, 2006)	X	X	X

**Potential Moderators**					

Sociodemographic characteristics	Age, sex, marital status, race, education, employment, income	Sociodemographic questionnaire	X	-	-
	Quality of relationship with primary lay caregiver	Dyadic Adjustment Scale (Spanier, 1976)	X	X	X
Clinical characteristics, post-transplant	Comorbidities	Charlson Comorbidity Index (Bernardini et al., 2004; Charlson et al., 1994)	X	X	X
	Symptoms after lung transplant	QLTP (DeVito Dabbs et al., 2004)	X	X	X
	Psychological distress	SCL-90-R (Anxiety and depression subscales; Derogatis, 1994; Rodin et al., 1991)	X	X	X
	Sleep quality	PSQI (Buysse et al., 1989)	X	X	X

*Note*. ACC/AHA = American College of Cardiology/American Heart Association Joint Committee on Clinical Practice Guidelines, BP = blood pressure, PSQI = Pittsburgh Sleep Quality Index, QLTP = Questionnaire for Lung Transplant Patients; SCL-90-R = Symptom Checklist 90-revised.

**Table 3:** Socio-demographic Characteristics at Baseline

Characteristic	Overall N = 88	LTGO n = 44	EUC n = 44	Test Statistic	df	p
Age, mean (range)^1^	56 (21–73)	58 (21–73)	55 (25–72)	1.10	86	0.27
Sex^2^				0.05	1	0.83
Male	48 (55%)	25 (57%)	23 (52%)			
Female	40 (45%)	19 (43%)	21 (48%)			
Marital Status^2^				1.18	2	0.55
Partnered	63 (72%)	32 (73%)	31 (70%)			
No Partner	24 (27%)	11 (25%)	13 (30%)			
Missing	1 (1.1%)	1 (2.3%)	0 (0%)			
Race^2^				3.74	2	0.15
White	78 (90%)	36 (84%)	42 (95%)			
Black	7 (8.0%)	5 (12%)	2 (4.5%)			
Other	2 (2.3%)	2 (4.7%)	0 (0%)			
Education^3^				-	-	0.64
Less than high school	7 (8.0%)	3 (6.8%)	4 (9.1%)			
High school grad	32 (36%)	14 (32%)	18 (41%)			
Some college	49 (56%)	27 (61%)	22 (50%)			
Employment^3^				-	-	>0.90
Working	4 (5.0%)	2 (4.9%)	2 (5.1%)			
Not Working	76 (95%)	39 (95%)	37 (95%)			
Income^2^				0.31	3	>0.90
<$20,000	13 (16%)	6 (15%)	7 (18%)			
$20,000–49,000	24 (30%)	12 (29%)	12 (30%)			
$50,000–99,999	28 (35%)	14 (34%)	14 (35%)			
>$100,000	16 (20%)	9 (22%)	7 (18%)			

1 Age is an ordinal variable, and a *t*-test was used.

2 Categorical variables marital status, race, and income used Pearson’s Chi-squared test.

3 Education and employment were tested with Fisher’s exact test as there were cells with <5 values.

**Table 4:** Raw Values for Primary and Secondary Outcomes per Assessment Time Point and by Treatment Group

Variable	Measure	Time	LTGO		EUC		Test Statistic	p

			n	Value	n	Value		
Physical function, mean (SD)	SGRQ^1^	Baseline	44	32 (19)	44	31 (20)	−0.12	.91
		3-months	38	23 (16)	39	25 (20)	0.65	.52
		6-months	40	22 (16)	37	24 (21)	0.54	.59
	Berg Balance^1^	Baseline	42	53.7 (2.8)	44	52.7 (4.3)	−1.26	.21
		3-months	31	54.6 (2.4)	34	54.1 (2.4)	−0.76	.45
		6-months	34	54.8 (2.1)	36	54.3 (2.8)	−0.72	.47
	Lower Body Strength (30-s STS)^1^	Baseline	41	9.6 (2.9)	29	9.8 (3.6)	0.21	.84
		3-months	30	10.5 (2.8)	35	10.4 (3.6)	−0.22	.82
		6-months	35	11.0 (3.2)	35	10.6 (3.9)	−0.47	.64

Physical activity, mean (SD)	Walking (Average steps/day)^1^	Baseline	18	3,616 (2,959)	18	3,012 (2,546)	−0.66	.51
		3-months	18	4,413 (3,381)	18	4,111 (4,514)	−0.23	.82
		6-months	18	4,529 (3,370)	18	3,529 (4,021)	0.81	.42
	Minutes spent in MVPA/day^1^	Baseline	41	11 (13)	41	9 (9)	−1.01	.32
		3-months	31	18 (23)	35	12 (12)	−1.17	.25
		6-months	29	13 (13)	32	12 (11)	−0.32	.75

Blood Pressure Control, n (%)	AHA Cat^2^	Baseline	44		44		6.09	.11
	1			9 (20%)		7 (16%)		
	2			10 (23%)		4 (9%)		
	3			17 (39%)		16 (36%)		
	4			8 (18%)		17 (39%)		
	AHA Cat^2^	3-months	43		44		5.97	.11
	1			12 (27%)		8 (19%)		
	2			10 (23%)		5 (12%)		
	3			12 (27%)		10 (23%)		
	4			10 (23%)		20 (47%)		
	AHA Cat^2^	6-months	42		40		1.50	.68
	1			11 (28%)		9 (21%)		
	2			7 (18%)		5 (12%)		
	3			7 (18%)		11 (26%)		
	4			15 (38%)		17 (40%)		

1 t-tests.

2 Pearson’s chi-squared.

*Note*. AHA = American Heart Association, MVPA = moderate-to-vigorous physical activity, SGRQ = St. George Respiratory Questionnaire, 30-s STS = 30-second Sit-to-Stand. For SGRQ and blood pressure lower scores indicate better function. For all other measures, higher scores indicate better functioning or activity. The number of participants for each specific timepoint and measure are displayed.

**Table 5:** Comparison of Change Scores for Outcomes Between 0–3 months and 0–6 months, by Treatment Group

Variable	Measure	Time	LTGO		EUC		Test statistic^3^	p

			n	Score	n	Score		
Physical Function	SGRQ^1^	0–3	44	−6 (12)	44	−5 (9)	0.75	.45
		0–6	38	−7 (8)	39	−6 (11)	0.21	.83
	Berg Balance^1^	0–3	42	0.7 (1.7)	43	0.5 (2.0)	−0.42	.67
		0–6	31	0.7 (1.7)	34	1.7 (3.3)	1.56	.12
	Lower Body Strength (30-s STS)^1^	0–3	41	0.3 (2.1)	29	1.0 (2.0)	1.21	.23
		0–6	30	1.1 (3.3)	35	1.5 (2.5)	0.53	.59

Physical activity	Walking (Average steps/day)^1^	0–3	18	798 (3,014)	18	1,099 (4,092)	0.25	.80
		0–6	18	913 (3,585)	18	517 (3,216)	−0.35	.73
	Minutes spent in MVPA/day^1^	0–3	41	7 (22)	41	2 (13)	−0.96	.34
		0–6	31	1 (13)	35	2 (7)	0.36	.72

Blood Pressure Stage^2^	Improved Same Worsened	0–3	44	17 (39%)	44	13 (30%)	0.81	.67
				14 (32%)		17 (40%)		
				12 (30%)		12 (30%)		
	Improved Same Worsened	0–6	43	13 (33%)	44	14 (33%)	1.49	.48
				11 (28%)		16 (38%)		
				16 (40%		12 (29%)		

1 Mean (SD).

2 n (%).

3 Wilcoxon rank sum exact test, Wilcoxon rank sum test, or Pearson’s Chi-squared test.

*Note*. MVPA = moderate-to-vigorous physical activity, SGRQ = St. George Respiratory Questionnaire, 30-s STS = 30-second Sit-to-Stand. The number of participants is reported for each time range and measure. The number includes only participants with data at both time points. Test statistics and p-values are for the analysis of between group differences.

**Table 6:** Results of Mixed Effects Models for Primary and Secondary Outcomes (N = 88)

Outcome	Predictor	Coeff	SE	Test Statistic	p	95% CI
SGRQ^1^	Time	−3.14	0.78	−4.03	<.001	(−4.67, −1.62)
	Group	0.77	4.46	0.17	.86	(−7.95, 9.49)
	Time × Group	−0.68	1.12	−0.61	.55	(−2.87, 1.51)

Berg Balance^1^	Time	0.84	0.18	4.63	<.001	(0.49, 1.20)
	Group	1.47	0.78	1.89	.06	(−0.04, 2.99)
	Time × Group	−0.44	0.25	−1.73	.09	(−0.94, 0.06)

Lower Body Strength (30-s STS)^1^	Time	0.66	0.21	3.19	0.001	(0.25, 1.06)
	Group	0.07	0.84	0.09	.93	(−1.56, 1.71)
	Time × Group	−0.10	0.29	−0.36	.72	(−0.66, 0.46)

Walking (Average steps/day)^1^	Time	258.44	388.20	0.67	.51	(−501.89, 1018.76)
	Group	239.15	1477.66	0.16	.87	(−2624.99, 3103.30)
	Time × Group	198.08	548.99	0.36	.72	(−877.18, 1273.34)

Minutes spent in MVPA/day^1^	Time	1.45	1.28	1.13	.26	(1.05, 3.97)
	Group	4.08	4.19	0.97	.33	(−4.09, 12.26)
	Time × Group	−0.45	1.77	−0.26	.80	(−3.93, 3.01)

Blood pressure stage^2^	Time	−0.10	0.21	−0.46	.65	(−0.51, 0.32)
	Group	−1.23	0.67	−1.83	.07	(−2.55, 0.09)
	Time × Group	0.23	0.30	0.76	.45	(−0.36, 0.81)

1 Linear mixed-effect model, test statistic is reporting t-values.

2 Generalized linear mixed-effects model, test statistic is reporting z-values.

*Note*: MVPA = moderate-to-vigorous physical activity, SGRQ = St. George Respiratory Questionnaire, 30-s STS = 30-second Sit-to-Stand. Parameters estimated via linear mixed effects models to accommodate missing data; this likelihood-based approach retains participants with incomplete longitudinal records under the Missing at Random assumption. All 88 participants were retained despite missing data.

**Table 7:** Raw Values for Potential Moderators/Mediators per Assessment Time Point and by Treatment Group

Type	Variable		LTGO		EUC			
					
		Month	N	Value^1^	N	Value^1^	Test statistic	p
**Moderator**	Questionnaire for Lung Transplants Patients (QLTP), total score, mean (SD)^2^	0	44	27 (9)	44	27 (12)	976	>.9
		3	38	25 (12)	37	28 (13)	789	.4
		6	37	24 (12)	40	27 (13)	789	.6
	Pittsburgh Sleep Quality Index (PSQI), poor sleep quality yes, n (%)^3^	0	44	31 (70%)	44	25 (57%)	11.2	.8
		3	38	24 (63%)	37	26 (70%)	21.0	.1
		6	37	27 (73%)	39	25 (64%)	15.5	.4
	Symptom Check List-90, Any psychological distress yes, n (%)^3^	0	44	16 (36%)	44	17 (39%)	0	.8
		3	37	9 (24%)	37	10 (27%)	0	.8
		6	37	7 (19%)	40	17 (43%)	3.9	.047
	Charlson Comorbidity Index, total score, mean (SD)^2^	0	44	5 (2)	44	5 (2)	626	.2
		3	44	6 (2)	44	6 (4)	676	.5
		6	44	7(3)	44	7 (3)	773	.7
	Dyadic Adjustment Score, total score, mean (SD)^2^	0	43	66 (8)	44	66 (6)	639	.4
		3	36	65 (8)	37	63 (9)	552	.5
		6	37	65 (7)	40	62 (11)	697	.7

**Mediator**	Self-Efficacy to Regulate Exercise, total score, mean (SD) 2	0	44	68 (20)	44	66 (22)	996	.8
		3	39	64 (23)	37	68 (21)	656	.5
		6	37	64 (23)	40	66 (22)	706	.7
	Adherence to Self-Monitoring, percent of days Fitbit worn, mean (SD)^2^	0	-	-	-	-	-	-
		3	18	83 (27)	18	80 (26)	165	>.9
		6	18	69 (40)	18	80 (35)	138	.4

1 Mean (SD) or n (%).

2 Wilcoxon rank sum test.

3 Pearson’s Chi-squared test.

*Note*. Fitbit use began after randomization; values recorded for 3- and 6-month timepoints. The number of participants for each specific timepoint and measure are displayed.

**Table 8:** Treatment Effects Among Subgroups with Scores in the Highest Quartiles for Adherence to Self-Monitoring and SERE

Subgroup	Outcome	Coeff	SE	Test Statistic	p	95% CI
High adherence to perform self-monitoring (percentage of days wearing Fitbit) LTGO: n = 5 EUC: n = 4	SGRQ^1^	−6.33	12.11	−0.52	.61	−29.40, 16.73
	Berg balance^1^	4.55	1.97	2.31	.03	0.84, 8.25
	Lower Body Strength (30-s STS)^1^	1.30	1.74	0.74	.47	−1.95, 4.54
	Walking (Average steps/day)^1^	2397	2729	0.88	.39	−2724, 7518
	Minutes spent in MVPA/day^1^	5.20	8.36	0.62	.54	−10.32, 20.74
	BP stage^2^	1.35	5.94	0.22	.82	−149.58, 243.79

High scores for Self-Efficacy to Regulate Exercise (SERE) LTGO: n = 11 EUC: n = 12	SGRQ^1^	−0.69	5.58	−0.12	.90	−11.43,10.06
	Berg balance^1^	1.18	0.95	1.24	.22	−0.66, 3.02
	Lower Body Strength (30-s STS)^1^	0.22	1.52	0.15	.88	−2.72, 3.16
	Walking (Average steps/day)^1^	−934	3355	−0.28	.78	−7279, 5409
	Minutes spent in MVPA/day^1^	9.22	11.21	0.73	.47	−13.39, 29.86
	Blood pressure stage^2^	−1.28	1.83	−0.70	.48	−22.28, 3.63

1 Linear mixed-effect model, test statistic is reporting t-values.

2 Generalized linear mixed-effects model, test statistic is reporting z-values.

*Note*. MVPA = moderate-to-vigorous physical activity, SERE = Self Efficacy for Exercise, SGRQ = St George Respiratory Questionnaire, 30-s STS = 30-second Sit-to-Stand.
